# 286. Infectious Complications and Antimicrobial Utilization in Hospitalized Patients with COVID-19

**DOI:** 10.1093/ofid/ofab466.488

**Published:** 2021-12-04

**Authors:** J Hunter Fraker, Vidhi Gandhi, Lan Duong, Jai Kumar, Princy N Kumar, Joseph G Timpone

**Affiliations:** 1 MedStar Georgetown University Hospital, Washington, District of Columbia; 2 Georgetown University Hospital, Potomac, Maryland; 3 Georgetown University School of Medicine, Washington, District of Columbia

## Abstract

**Background:**

Hospitalized patients with COVID-19 have created increased demands on health care infrastructure and resources. Bacterial and fungal infections have been reported and have increased the need for antimicrobial utilization. We performed a retrospective chart review to characterize bacterial infections and antibiotic utilization during the COVID-19 surge at our tertiary care center.

**Methods:**

All patients diagnosed with COVID-19 using SARS-CoV-2 PCR admitted to MedStar Georgetown University Hospital from 01Mar2020 through 31Aug2020 were included in the analysis. Data was collected on hospital-wide antimicrobial utilization [mean days of therapy per 1000-patient-days (DOT)] during the 6-month surge and was compared to antimicrobial utilization during a 6-month period that preceded the COVID-19 surge. Clinical and microbiological data and patient outcomes were also collected and analyzed.

**Results:**

A total of 238 patients met eligibility criteria during the observation period, of which 25.6% (n = 61) developed a bacterial, fungal, or viral co-infection. Culture-positive bacterial complications were seen in 21.8% (n = 52) with 32.8% (n = 20) having a multidrug resistant organism (MDRO). There was a statistically significant difference between COVID-19 patients with co-infection and those without for intubation (p < 0.001), vasopressor use (p < 0.001), and renal replacement therapy (p = 0.001). COVID-19 patients with co-infections had a longer mean length of stay (21.9 days vs 13.5 days, p < 0.001) and greater mortality (32.8% vs 20.6%, p = 0.006) compared to those without a co-infection, respectively.

Mean antimicrobial utilization for the entire hospital population was 790.6 DOT during the COVID surge compared to 928.7 DOT during a 6-month period preceding the COVID surge (p < 0.001). For all COVID-19 patients, antimicrobial utilization was 846.9 DOT; however, this increased to 1236.4 DOT for COVID-19 patients with co-infections.

Table 1. Demographics

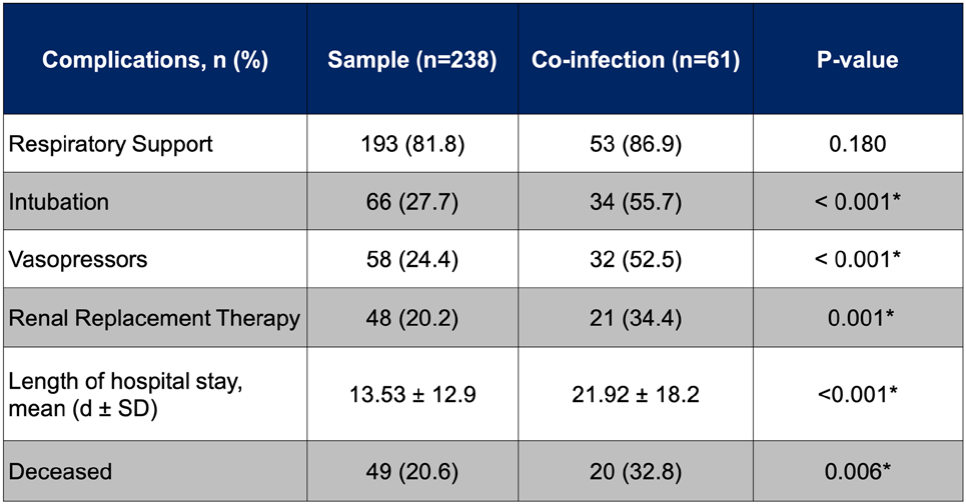

Table 2. Antimicrobial Utilization in COVID-19 Patients

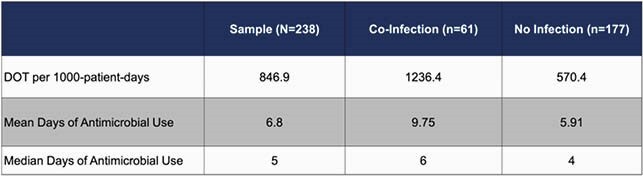

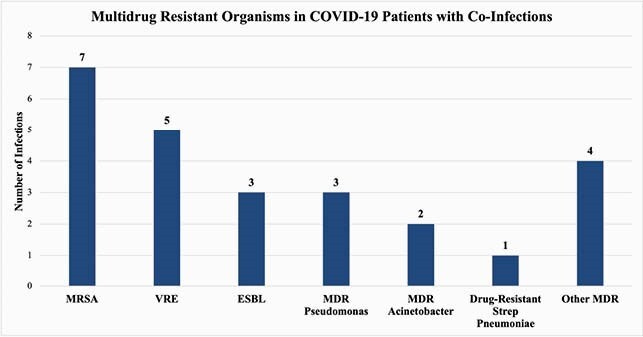

**Conclusion:**

Although hospital-wide antimicrobial utilization had decreased during the COVID surge, COVID-19 patients with co-infections demonstrated a disproportionate use of antimicrobial agents as well as ICU resources. As MDRO infections were relatively common, antimicrobial stewardship should be prioritized in the COVID-19 population.

**Disclosures:**

**Lan Duong, Pharm.D.**, **Astra Zeneca** (Shareholder)**Eli Lilly & Co.** (Shareholder)**Gilead Sciences, Inc.** (Shareholder)**Merck & Co.** (Speaker’s Bureau)**Moderna, Inc.** (Shareholder)**Novavax, Inc.** (Shareholder)**Sarepta Therapeutics** (Shareholder)**Thermo Fisher Scientific** (Shareholder) **Princy N. Kumar, MD**, **AMGEN** (Other Financial or Material Support, Honoraria)**Eli Lilly** (Grant/Research Support)**Gilead** (Grant/Research Support, Shareholder, Other Financial or Material Support, Honoraria)**GSK** (Grant/Research Support, Shareholder, Other Financial or Material Support, Honoraria)**Merck & Co., Inc.** (Grant/Research Support, Shareholder, Other Financial or Material Support, Honoraria)

